# Clinical features and outcomes of male patients with lymphangioleiomyomatosis: A review

**DOI:** 10.1097/MD.0000000000032492

**Published:** 2022-12-30

**Authors:** Haoyu Zhang, Zhigang Hu, Sufei Wang, Kanhao Wu, Qiaoyu Yang, Xinyu Song

**Affiliations:** a Department of Respiratory and Critical Care Medicine, the first College of Clinical Medicine science, China Three Gorges University, Yichang, People’s Republic of China.; b Department of Respiratory and Critical Care Medicine, Yichang Central People’s Hospital., Wuhan, Hubei, China; c Department of Respiratory and Critical Care Medicine, NHC Key Laboratory of Pulmonary Diseases, Union Hospital, Tongji Medical College, Huazhong University of Science and Technology, Wuhan, Hubei, China.

**Keywords:** characteristic, LAM, male, outcomes, sporadic LAM, TSC-LAM

## Abstract

Lymphangioleiomyomatosis (LAM) is a rare disease involving multiple systems, which is divided into sporadic LAM (S-LAM) and tuberous sclerosis complex-LAM, mostly affecting women who are in childbearing age stage. Data on male patients are limited and scattered. Therefore, it is necessary to conduct a systematic review to investigate the clinical features, diagnosis, treatment, and outcomes of LAM in male.

**Methods:**

We performed a literature review by searching for all the published reported cases of LAM in male during the past 35 years (April 1986–October 2021).

**Results:**

36 male patients described in 26 references were included in this article. The median age of onset was 34 years (interquartile range: 1–79). The most common initial manifestations were cough, dyspnea, respite, and hemoptysis, with pulmonary complications such as pneumothorax and chylothorax. Five patients (13.9%) were asymptomatic at admission. Nearly half of the 36 male patients had thin-walled air-filled cysts that were visible throughout both lungs. Considering the abovementioned atypical clinical features, misdiagnosis was committed in 8 patients (22.2%). In addition, patients with tuberous sclerosis complex lymphangioleiomyomatosis often have no pulmonary manifestations at onset but present multiple extrapulmonary manifestations and have higher rates of renal angiomyolipomas than patients with S-LAM (*P* < 0.01). Eventually, 4 patients with S-LAM eventually died.

**Conclusion:**

Physicians should increase the awareness of LAM in male. Early monitoring of various systems should be recommended to ensure early management and active follow-up. Tuberous sclerosis complex patients should immediately be tracked for the onset of LAM disease to improve prognosis.

## 1. Introduction

Lymphangioleiomyiomatosis (LAM) is a rare disease with diffuse pulmonary cystic degeneration and multisystem involvement, mostly affecting women who are in childbearing age stage, but rarely appears in male patients.^[1]^ Currently, LAM is estimated to affect more than 7.8 per million women,^[2]^ but the prevalence of LAM in male is lacking. Two subtypes have been identified: sporadic (S-LAM) and tuberous sclerosis complex (TSC-LAM) that are associated with LAM. The occurrence of patients with TSC-LAM is caused by mutations in the TSC1 or TSC2 genes.^[3]^ LAM occurs more frequently in patients with S-LAM, and patients with S-LAM can be caused by mutations of the TSC2 gene.^[4]^ Mutations in these genes result in the dysregulation of the mammalian target of the rapamycin mTOR pathway.^[5]^

LAM is a chronic progressive disorder. Clinical manifestations include dyspnea, cough, hemoptysis, and respite, with pulmonary complications such as pneumothorax and chylothorax. Cases from asymptomatic patients detected incidentally to severe pulmonary impairment have been also reported in men and women.^[6,7]^ TSC is an autosomal dominant disorder, which affects multiple organ systems, including the central nervous system, the skin, the eye, the kidney, and the lung. As the disease progresses, patients with TSC may develop disease with LAM.^[8]^ The lower prevalence 13% of male with TSC had cystic lung changes consistent with LAM compared with 42% in female patients with TSC.^[9]^ Therefore, cystic changes in the lungs of patients with TSC should be screened immediately for LAM disease. S-LAM is another form of LAM disease, which is common in the general population. According to the accidental discovery on computed tomography (CT) of the abdomen or the lungs, 12% of patients with S-LAM have cystic lung or abdomen changes.^[10]^ Thus, patients with S-LAM are often found accidentally in extrapulmonary organs. Previous studies that compared patients with TSC-LAM and S-LAM found no difference in pulmonary manifestations.^[11]^ Published data from the National Heart, Lung and Blood Institute (NHLBI) LAM Registry showed that TSC-LAM was milder. However, differences were not observed in the progression to death between these groups.^[12]^ LAM in male was firstly described in 1986 by Fairfax.^[13]^ Subsequently, several rare reports of misdiagnosis of LAM disease have been recorded in men.^[14,15]^ Unfortunately, when diagnosed incorrectly, this condition may result in respiratory failure. In the early stages of disease, patients do not seek medical attention or might be neglected by medical professionals, resulting in poor outcomes.

Studies on the single cell analysis showed that a unique population of LAMCORE cells was identified in lung and uterus of patients with LAM, sharing close transcriptomic identity, and they think that the uterus may be the origin of the cells driving LAM in the lungs.^[16]^ However, we have evidence in the literature that LAM does occur in male. Therefore, this subtype (male with LAM) needs to be investigated. In this systematic review, we summarize the clinical features, diagnosis, treatment, and outcomes of LAM in male. The differences between patients with TSC-LAM and S-LAM were determined to increase the awareness of LAM in male by physicians and help in the early recognition.

## 2. Materials and methods

### 2.1. Information sources and search strategies

A retrospective analysis of all reported cases of LAM in male from 1986 to 2021 was conducted. We performed this research using the following keywords: “Lymphangiomyomatosis” or “LAM” and “male” or “man.” A systematic literature search was performed using PubMed, CNKI, and Wanfang data. We reviewed the references of the collected articles to ensure that all patients included in this study fulfilled the current diagnostic criteria for definite LAM. Medical records of these patients were reviewed to extract the following data: age, sex, smoking history, TSC–LAM, clinical presentation, imaging studies, results of the biopsy, diagnoses, misdiagnosis, treatment, and outcomes.

### 2.2. Data collection

Standardized Excel tables were used to extract data from all eligible articles, and such information is included: basic information of studies: publication year, author’s name, title, countries, age, gender, smoking status, family history, with TSC status, age at diagnosis of LAM. Clinical manifestations: the Initial clinical appearance, typical clinical appearance during the course of the disease. Imaging manifestations: pulmonary CT findings, pulmonary thin-walled cysts location, number and maximum diameter, other relevant abnormal findings. Diagnostic technology: biopsy, abnormal CT, Chest X-ray, vascular endothelial growth factor-D levels, and other relevant methods. Misdiagnosed diseases: angiomatosis, chronic bronchitis, lung cancer, or other diseases. Treatment methods: surgery, sirolimus, hormonal manipulation, lung transplantation, interferon treatment, and no treatment. Follow-up outcome: alive, improved, stable or unchanged, worsened or died, missing information.

### 2.3. Definitions

According to the LAM diagnostic classification criteria published in 2016^[17]^ and LAM diagnostic guide published in 2017.^[18]^ 36 patients of LAM were classified as follows: characteristic or high-resolution computed tomography (HRCT) and typical pathological features on lung biopsy or biopsy of other locations in 35 patients. One patient with TSC who was not suitable for biopsy was diagnosed based on serum biomarker vascular endothelial growth factor-D  > 800 pg/ml and typical lung CT findings. Furthermore, the diagnosis of LAM was based on unequivocally reported case reports.

### 2.4. Statistical analysis

Demography, clinical characteristics, treatment modalities, and outcomes of all published cases were extracted from the full-text articles. Data were included in a specific database and analyzed. Categorical variables were expressed as counts and percentages; continuous variables were presented as median and interquartile range (IQR) values. We defined the follow-up duration as the time elapsing from the initial diagnosis of LAM to the time of loss to follow-up or death. Statistical analysis was performed using Microsoft Excel (version 2010) and IBM SPSS statistics software (version 22). *P* values < 0.05 (2-sided) were considered statistically significant.

## 3. Results

### 3.1. Patient characteristics

Thirty-three case reports were published, including 58 participants. Duplicating case reports and incomplete cases were removed. Finally, 26 case reports and 36 participants were included in the pooled analysis.^[7,13–15,19–40]^ As shown in Table 1, all screened patients were male. The median age of the participants was 34 years (IQR: 1–79). The participants were from different countries, including East Asia (n = 20), South Asia (n = 1), North America (n = 10), Europe (n = 4), and Oceania (n = 1). Among these 36 patients, 9 patients had definite TSC. The most common symptoms were dyspnea (n = 10), cough (n = 8), hemoptysis (n = 4), and respite (n = 4). The most common pulmonary complications included pneumothorax (n = 10) and chylothorax (n = 3). Twelve patients were misdiagnosed, and the median time of misdiagnosis was at least 1 year. The patients were usually misdiagnosed as angiomatosis, chronic bronchitis, lung cancer, or other diseases, and the frequency of misdiagnosis tended to be higher in the group S-LAM (37%) than in the group with TSC (22.2%). Diagnosis was mostly based on lung CT combined with pathological examination. The common treatment method was surgery (n = 14). Follow-up results revealed that 4 patients worsened or died, and 23 patients were alive. The median time from diagnosis to death was 2.6 years (IQR: 2 months–7 years). More detailed characteristics are summarized (see Table S1, http://links.lww.com/MD/I232, Digital Supplemental Content, which literature review (1986–2021) Summary of patients detailed characteristics).

**Table 1:** Literature review (1986–2021) Summary of patient characteristics.

Characteristic	No. of patients	Percent (%)
Age, yr, median (range)	34 (1 mo–79 yr)	NA
Country		
East Asia	20	55.60
South Asia	1	2.80
North America	10	27.80
Europe	4	11.10
Oceania	1	2.80
Smoking status		
Never	4	11.10
Current	3	8.30
Unknown	29	80.60
TSC-LAM	9	25
Sporadic LAM	27	75
Clinical manifestations		
Dyspnea as the initial symptom	6	16.70
Dyspnea at presentation	10	27.80
Cough as the initial symptom	7	19.40
Cough at presentation	8	22.20
Hemoptysis as the initial symptom	3	8.30
Hemoptysis at presentation	4	11.10
Respite as the initial symptom	4	11.10
Respite at presentation	4	11.10
Abdominal pain as the initial symptom	5	13.90
Abdominal pain at presentation	5	13.90
Asymptomatic	5	13.90
Complications		
Pneumothorax	10	27.80
Chylothorax	3	8.30
Renal AMLs	7	19.40
Others AMLs	6	16.70
Investigations (misdiagnosis status)		
Angiomatosis	3	5.60
Chronic bronchitis	2	5.60
Lung cancer	3	8.30
Other diseases	4	11.10
Diagnosis method		
Biopsy (pathology)	35	97.20
Chest CT	25	69.40
Chest X–ray	8	22.20
Others method	2	5.60
Treatment		
Surgery	14	38.90
Sirolimus	2	5.60
Hormonal manipulation	3	8.30
Lung transplantation	1	2.80
Interferon treatment	2	5.50
No special treatment	17	44.40
Follow-up data		
Alive	23	63.90
Improved	9	25
Stable/unchanged	14	38.90
Worsened/died	4	11.10
Unknown	9	25

AMLs = angiomyolipomas, CT = computed tomography, LAM = lymphangioleiomyomatosis, Mo = Month, NA = not available, TSC = tuberous sclerosis complex, Yr = year.

### 3.2. Clinical manifestations of LAM in male

All cases specifically described the clinical characteristics of 36 male patients with LAM. Cough was the most common initial manifestation of LAM in male (Table 1) and was seen in more than 19.4% of patients. Other initial manifestations included dyspnea (6/36, 16.7%), abdominal pain (5/36, 13.9%), respite (4/36, 11.1%), and hemoptysis (3/36, 8.3%). During the disease, the most common symptoms of LAM in male were dyspnea (10/36, 27.8%), followed by cough (8/36, 22.2%), respite (4/36, 11.1%), and hemoptysis (4/36, 11.1%). And the most common pulmonary complications were pneumothorax (10/36, 27.8%) and chylothorax (3/36, 8.3%). The numbers and location of pneumothorax in these patients during the course of the disease were also mentioned. Over 5 patients had more than 3 pneumothoraxes, and 7 patients showed unilateral involvement.

The clinical features of patients with TSC-LAM and S-LAM were compared (Table 2). Nearly 5 TSC patients (55.6%) developed progressive dyspnea during the course of the disease. Patients with TSC were more likely to present gradual onset of dyspnea. However, no significant difference was observed in the onset of dyspnea between patients with TSC-LAM and S-LAM (*P* = .08). 3 patients with TSC-LAM (33.3%) were asymptomatic, and 6 patients were mildly symptomatic on admission. Six patients with TSC-LAM had renal angiomyolipoma (AML)s at an early stage. TSC-LAM patients were likely to have renal AMLs compared with those with S-LAM (*P* < .01). Patients with TSC-LAM having renal AMLs had multiple lesions, bilateral involvement, and more than one extrapulmonary region involved, including skin, liver, kidney or intracranial lesions.

**Table 2:** Symptoms and performance characteristics, research and outcomes of patients with TSC-LAM and Sporadic LAM in male.

	TSC-LAM	Sporadic LAM	*P* value
	n = 9	n = 27	
Clinical manifestations			
Dyspnea during course of disease	5 (55.6%)	5 (18.5%)	0.08
Cough during course of disease	2 (22.2%)	6 (22.2%)	1.00
Hemoptysis during course of disease	2 (22.2%)	2 (74.0%)	0.26
Respite during course of disease	0 (0%)	4 (14.8%)	0.55
Abdominal pain during course of disease	1 (11.1%)	4 (14.8%)	1.00
Asymptomatic	3 (33.3%)	2 (7.4%)	0.09
Complications			
Pneumothorax	4 (44.5%)	6 (22.2%)	0.22
Renal AMLs	6 (66.7%)	1 (3.7%)	<0.01
Immunohistochemical			
Spindle cells	4(44.4%)	13 (48.1%)	1
Lining cells	2 (22.2%)	11 (40.7%)	0.44
Investigations (misdiagnosis status)	2 (22.2%)	10 (37%)	0.68
Follow-up data			0.57
Alive	9 (100%)	23 (85.2%)	
Died	0	4 (14.8%)	

Values are presented as number with percentage (%). AMLs = angiomyolipomas, LAM = lymphangioleiomyomatosis, TSC = tuberous sclerosis complex.

### 3.3. Imaging examination and clinicopathologic features of LAM in male

Twenty-five patients (69.4%) were examined by chest CT, and abnormalities such as thin-walled air-filled cysts throughout the lungs (15/25, 60%) and high-density nodules (9/25, 36%) were detected in 24 patients. The majority of pulmonary thin-walled cysts were detected in patients with TSC-LAM (7/9, 77.8%). The diameters of air-filled cysts were mostly below 10 mm. Abnormal findings were found on abdominal CT or kidney and liver ultrasonography in 10 patients (27.8%). Eight patients (22.2%) underwent chest X-ray and showed diffuse interstitial infiltrates. Two patients were in abnormal condition based on colonoscopy tests.

Among the 36 patients, 1 patient did not have pathological examination because of poor lung function.^[20]^ Thus, 35 patients had pathological examination results. Thirty-three patients showed the characteristics of cystic lesion, multiple immature smooth muscle cells, and abnormal proliferation of perivascular epithelial cells. Two patients provided pathological diagnostic results without detailed descriptions. The immunohistochemical findings are shown in (Table 3). Pathological specimens of common sources included lung biopsy (23/35, 65.7%), transbronchial lung biopsy (1/35, 2.9%), thoracotomy (4/35, 11.4%), thoracoscopy (2/35, 5.7%), and mesenteric biopsy (3/35, 8.6%). In immunohistochemistry examination, positives results were obtained in human melanoma black 45 (10/25, 40%), smooth muscle actin (19/25, 76%), estrogen receptor (4/25, 16%), Pathology results showed spindle cells in 4 patients with TSC-LAM (44.4%) and 13 patients with S-AM (48.1%). No substantial differences in pathology were found between patients with TSC-LAM and those with S-LAM (Table 2).

**Table 3:** Immunohistochemical findings of LAM in male.

Specimen source	No. of patients	No. of positive/negative	
Lung biopsy	23 (65.7%)	Spindle cells	
		Vimentin	6/0
TBLB	1(2.9%)	Desmin	17/2
		Estrogen receptor	4/15
Thoracotomy	4(11.4%)	HMB-45	10/18
		SAM	17/2
Thoracoscopy	2(5.7%)	Lining cells	
		Vimentin	8/0
Mesenteric biopsy	3(8.6%)		
Others location	2(5.7%)		

Values are presented as number with percentage (%).HMB-45 = human melanoma black 45, LAM = lymphangioleiomyomatosis, SMA = smooth muscle actin, TBLB = transbronchial lung biopsy.

### 3.4. Treatment and outcomes of LAM in male

Nineteen of these patients had detailed treatment records. Nine (25%) patients were on hormonal therapy and sirolimus. Fifteen (41.7%) patients underwent surgery, including 1 patient with lung transplantation, and 14 with pneumonectomy. The 17 remaining patients (44.4%) did not receive special treatment after being diagnosed with LAM. The information on survival status was available in 27 patients (75%), whom were all followed for at least 2 months, and the longest follow-up duration was 7 years. Nine patients had improved symptoms and better outcome. Fourteen patients had no progression or remained stable. Eventually, 4 patients with S-LAM died because of disease exacerbation, including 3 patients who underwent lung surgery and 1 patient without special treatment. The death of the 4 patients can be attributed to progressive chylothorax, respiratory failure, and massive hemoptysis. The minimum age of deceased patients was 1 month, and the maximum age was 76 years old. The mean time interval from diagnosis to death was 2.6 years (IQR: 2 months–7 years).

## 4. Discussion

The clinical spectrum of LAM is broad, involving multiple systems, including the lungs, lymphatic vessels, kidneys, and liver, and this condition is especially rare in men. Frequent misdiagnosis cases can present a serious public health threat. However, data on male patients are limited and scattered. In the present study, 36 patients from published case literature were investigated.

The typical clinical manifestations of male with LAM are similar to those of most females with LAM, including cough, dyspnea, hemoptysis, and respite. The most common complications include pneumothorax, chylothorax, and renal AMLs. The underlying pathological mechanism is that the hyperactivation of the target of rapamycin (mTOR) pathway leads to dysregulated cell proliferation. This process may ultimately promote LAM cells into the circulation, resulting in their expression in various organs.^[41]^ Moreover, the initial symptom of LAM in male is cough, and the symptoms are mild. However, women tend to have dyspnea as the initial symptom.^[42]^ Sex differences may also be present in the onset characteristics. This difference may be the underlying effect of female estrogen receptors and progesterone receptors on the progression of LAM.^[43]^ Considering that LAM in male has mild and atypical symptoms at the onset, this condition tends to be ignored. Further investigation of sex differences in LAM is necessary. In addition, we investigated differences in clinical characteristics between patients with TSC-LAM and S-LAM. Based on the results, compared with patients with S-LAM, patients with TSC-LAM are likely to accompany AMLs and involve more than 1 extrapulmonary region. Hancock and his colleagues^[44]^ performed a retrospective study over 58 years (1939–1997) in 444 patients, including 9 male patients. Results show that in addition to frequent comorbidities with the involvement of bilateral AMLs, patients with TSC-LAM were more asymptomatic at onset and were more likely to develop a gradual onset of dyspnea during the course of the disease than those with S-LAM. Rebaine and his colleagues^[45]^ also compared the clinical characteristics between the 2 groups and found that patients with TSC-LAM had milder symptoms at the onset, but this condition was difficult for clinicians to detect. However, no significant difference was observed in the consistency with our pooled results. This finding was obtained possibly because the majority of cases reported were not genetically analyzed, thus underestimating the prevalence of patients with TSC. The above results have several explanations. First, TSC1 and TSC2 pathogenic mutations can lead to hemangioma formation through constitutively activated pathways of the mTOR pathway.^[46]^ Second, the lower frequency of lymphatic involvement observed in patients with TSC-LAM may account for their mild pulmonary symptoms.^[47]^

The average age at the onset of LAM in men is similar to that of most female with LAM.^[48]^ Younger male patients are likely to develop LAM. However, a longer period of delay is observed between the onset and diagnosis in male. A possible interpretation is that LAM was under-recognized and ignored in male. In this study, the majority of patients were diagnosed by HRCT and pathology. HRCT was helpful in determining the cystic nature of the lesions and the extent of the disease. Two kinds of cystic lung lesions were found in male, including commonly diffuse air sacs and nodule lesions. The nodules lesions may have been caused by the increase in immature smooth muscle cells and perivascular epithelial cells. In the present study, the majority of the diameters of air-filled cysts were below 10 mm in both patients with TSC-LAM and S-LAM. However, the diameters of air-filled cysts varied from 2 mm to more than 2 cm in female, and more and larger cysts were found in women than in men.^[49]^ In addition, Cormack^[50]^ found that patients with cyst size > 5 mm are more likely to have recurrent pneumothorax than those with cyst size < 5 mm. The maximum diameters in 2 patients exceeded 5mm and had 5 times the incidence of pneumothorax. At present, the existence of TSC provides the basis for the clinical diagnosis of LAM by characteristic HRCT. The existence of TSC in this study can provide a basis for characteristic HRCT diagnosis of LAM, but if patients with TSC are not screened early for all organs, diagnosis may be delayed. Therefore, patients with TSC-LAM are challenging to diagnose.

Currently, the rarity of patients with LAM in male limits the therapeutic measures, and several studies have assessed long-term treatment effects. Considering the limited understanding of LAM disease in male, traditional surgical resection is generally more selected when the lungs are abnormal in imaging, and the diagnosis is unclear. However, surgical resection may be unacceptable and unnecessary for men with milder symptoms. The curative effect of hormone treatment for LAM has not been supported among the patients with TSC-LAM or S-LAM. This study results showed that 3 patients remained stable after hormone therapy in the short-term. This result may have been obtained, because the patient’s early symptoms were milder and the course of deterioration was slow. Whether hormone therapy can also be targeted at men remains unclear. Thus, hormone therapy should be given with caution in male. The mTOR inhibitors remain the first-line drug for LAM in both men and women.^[51]^ Based on the current findings, renal hemangioma volume was reduced in 2 patients with TSC-LAM who were treated with sirolimus. Furthermore, 1 male patient with advanced stage survived for 7 years after lung transplant. However, a median survival time of 12 years has been recorded after transplantation in female patients.^[52]^ Whether male patients have a shorter survival time after lung transplantation needs to be confirmed in future studies with large sample size. Further, we investigated outcomes based on reported information. Our results showed that 4 patients with S-LAM died. Regrettably, most patients with TSC-LAM were lost to follow-up, and the true number of deaths is hard to determine. Data published by the National Heart, Lung and Blood Institute (NHLBI) LAM Registry showed no significant difference in death progression between patients with TSC-LAM and those with S-LAM for woman.^[12]^ Unfortunately, there are no literatures reported survival differences between those patients with TSC-LAM and S-LAM groups in male.

This study had some potential limitations. Firstly, our research data is limited to published articles. Secondly, the total number of male patients was relatively small, so the interpretation of our findings might be limited due to sample size. Thirdly, the response to treatment varies greatly among different patients, and treatment methods may also be an important factor affecting prognosis. Fourth, the actual number of TSC patients in this study may be underestimated, and there might potential selection bias. Finally, the imbalanced population size between the 2 groups may also have limited our analysis. Therefore, large cohort studies are likely to be required.

## 5. Conclusion

LAM in male has mild onset symptoms and is often overlooked and misdiagnosed. Meanwhile, treatment options are limited. For patients with TSC, early attention should be paid to various systematic examinations and active follow-up to ensure the early detection of LAM disease and improve prognosis as soon as possible. In terms of treatment, mTOR inhibitors are still recommended as first-line drugs for LAM in male, but the efficacy evaluation is limited because of the lack of case data in male. In addition, sex characteristics should be considered, and it remains to be determined whether there are gender differences in LAM disease have not been determined in some aspects (e.g., clinical characteristics and outcomes).

## Author contributions

**Conceptualization:** Haoyu Zhang.

**Data curation:** Haoyu Zhang, Sufei Wang, Kanhao Wu, Qiaoyu Yang.

**Funding acquisition:** Xinyu Song.

**Investigation:** Haoyu Zhang, Zhigang Hu.

**Methodology:** Haoyu Zhang, Sufei Wang.

**Resources:** Xinyu Song.

**Writing—original draft:** Haoyu Zhang.

**Writing—review and editing:** Haoyu Zhang, Zhigang Hu, Sufei Wang, Xinyu Song.

## Supplementary Material
