# Supplementary material for: Clinical features and outcomes of male patients with lymphangioleiomyomatosis: A review
Source: Medicine (Baltimore). 2022 Dec 30;101(52):e32492. doi: 10.1097/MD.0000000000032492 (PMC9803497; doi:10.1097/MD.0000000000032492)
Supplement: Supplementary file 1 [file medi-101-e32492-s001.pdf]

Table S1 Literature review (1986 to 2021) Summary of patients detailed characteristics

**Author(reference) Year**	**Country**	**Age Sex**	**Smoking**	**Form**	**Presentation**	**Imaging presentation**	**Diagnostic technology**	**Misdiagnosed disease**	**Treatment**	**Course**
Fairfax et al 1986	Europe London	51yr M	Unknown	S-LAM	Chylothorax	NA	Thoracotomy	NA	NA	Died of progressive chylothorax 22 mo later.
Kang et al 1991	East America Korea	22yr M	Unknown	S-LAM	Dyspnoea Pneumothorax (n=3)	CT: thin-walled air-filled cysts(upper lung) throughout both lungs	Lung biopsy Pathology	NA	NA	Lost to follow-up.
Tazelar et al *1993*	North America USA	1mo M	Unknown	S-LAM	Respite Chylothorax(n=3)	Chest radiograph bilater Pleural a pericadial effusions interstitial	Mesenteric biopsy pathology	NA	Lung transplantion	Progressive respiratory compromise died at 7 yr.
	North America	11mo M	Unknown	S-LAM	Cough	diffuse interstitial	Lung tissue pathology	NA	NA	Alive 2.5 yr later.
	North America USA	3yr M	Unknown	S-LAM	Respite	Cardiomegaly interstitial infiltrates	Lung tissue pathology	Angiomatosis	Pneumonectomy	Died of massive Hemoptysia 1.5 yr later.
	North America USA	5yr M	Unknown	S-LAM	Asthma history of poland's syndrome	Interstitial pneumonia	Lung tissue pathology	NA	NA	Alive 6 mo later.
	North America USA	7yr M	Unknown	S-LAM	Respite	Diffuse interstitial	Lung tissue pathology	NA	NA	Alive 6 mo later.

Table S1 (Continued)

Author(reference)<br>Year	Country	Age<br>Sex	Smoking	Form	Presentation	Imaging presentation	Diagnostic technology	Misdiagnosed disease	Treatment	Course
Tazelar et al<br>1993	North<br>Americal<br>USA	16yr<br>M	Unknown	S-LAM	Shortness of breath	Diffuse<br>interstitial infiltrare<br>pleural effusions	Lung biopsy<br>pathology	NA	NA	Alive and well<br>1.5 yr later.
	North<br>Americal<br>USA	33yr<br>M	Unknown	S-LAM	Asthma<br>since childhood	Bibasilar interstitial<br>infiltrates	Lung biopsy<br>pathology	NA	Tamoxifen	Alive and well<br>2yr later.
Bowen et al<br>1997	Oceania<br>Australia	8 yr<br>M	Never	TSC-LAM	Dyspnoea<br>Pneumothorax(n=1)<br>Renal AMLs	Multiple cysts<br>(left chest)<br>with collapse	Thoracotomy<br>pathology	NA	Hormonal<br>manipulation	Lost to<br>follow-up.
Yu et al<br>1997	East<br>Asia<br>China	34yr<br>M	Unknown	S-LAM	Abdominal pain	US：right liver lobe<br>and right	Mesenteric biopsy<br>Pathology	NA	NA	Lost to<br>follow-up.
Aubry et al<br>2000	North<br>Americal<br>USA	39yr<br>M	Current	TSC-LAM	Dyspnoea<br>Hemoptysis<br>Renal AMLs	CT: diffuse cystic<br>lesions (both lungs)	Lung biopsy<br>pathology	NA	NA	Aive and well 9 mo<br>Symptoms improved.
Guo-Kun et al<br>2001	East<br>Asia<br>China	25yr<br>M	Unknown	S-LAM	NA	CT: diffuse cystic<br>lesions (both lungs)<br>within 10mm in<br>diameter	Lung biopsy<br>pathology	NA	NA	NA
Kim et al<br>2003	East<br>Asia<br>China	47yr<br>M	Unknown	TSC-LAM	Abdominal pain<br>Dyspnoea<br>Renal AMLs	CT: pulmonary cystic<br>lesions up to 10mm	Lung biopsy<br>pathology	NA	NA	Lost to follow-up.

Table S1 (Continued)

Author(reference) Year	Country	Age Sex	Smoking	Form	Presentation	Imaging presentation	Diagnostic technology	Misdiagnosed disease	Treatment	Course
Pileri et al 2004	North Americal USA	25yr M	Unknown	TSC-LAM	Asymptomatic	CT: pulmonary cystic lesions up to 10mm	Lung biopsy pathology	NA	NA	alive 20 mo later
Fiore et al 2005	Oceania Italy	37yr M	Never	S-LAM	Abdominal pain	CT: abdominal mass	Mesenteric biopsy pathology	Mesenteric lymphoma	Hormonal manipulation	alive 48 mo later
Wang et al 2005	East Asia China	25yr M	Unknown	S-LAM	Abdominal pain	Colonoscopy proliferation of smooth muscle cells	Mesenteric biopsy pathology	NA	NA	Lost to follow-up
Schiavina et al 2007	Oceania Italy	37yr M	Never	S-LAM	Pneumothorax(n=1)<br>Liver AMLs	CT: diffuse cystic lesions (both lungs) 0.5-1.5cm in diameter	Mesenteric biopsy pathology	NA	NA	alive 3yr later
Steinacher et al 2009	Oceania Paris	30yr M	Unknown	S-LAM	Chylothorax<br>Cough<br>Dyspnoea<br>Hemoptysis<br>Liver AMLs	CT: diffuse cystic lesions (both lungs) few millimeters in diameter	Thoracotomy pathology	Pulmonary bullae	Lung cystectomy octreotide	alive 3yr later
Zhou et al 2013	East Asia China	17yr M	Unknown	S-LAM	Cough<br>Dyspnoea<br>Pneumothorax(n=2)	CT: diffuse cystic lesions (both lungs)	Symptom<br>CT imaging	Chronic bronchitis	NA	Lost to follow-up
Wang et al 2013	East Asia China	76yr M	Unknown	S-LAM	Asymptomatic	CT: diffuse cystic lesions (mainly left upper lung)	Lung biopsy pathology	Pulmonary bullae	Pneumonectomy	Died of progressive dyspnea 2 mo later

Table S1 (Continued)

Author(reference)<br>Year	Country	Age<br>Sex	Smoking	Form	Presentation	Imaging presentation	Diagnostic technology	Misdiagnosed disease	Treatment	Course
Li et al<br>2014	East<br>Asia<br>China	76yr<br>M	Unknown	S-LAM	Cough<br>Pneumothorax(n=1)	CT: high-density<br>nodules (both lungs)	Lung biopsy<br>pathology	Multiple lung<br>metastases	NA	Alive 6 mo<br>later
Wakida et al<br>2015	East<br>Asia<br>Japan	17yr<br>M	Unknown	TSC-LAM	Pneumothora(n=1)<br>Renal AMLs	CT: diffuse Cystic<br>lesions (both lungs)	Lung biopsy<br>pathology	NA	Sirolimus	Alive 1yr later
He et al<br>2016	East<br>Asia<br>China	73yr<br>M	Unknown	S-LAM	Dyspnoea	CT: high-density<br>nodules (both lungs)	Mesenteric biopsy<br>pathology	NA	NA	Alive in<br>the short term
Wang et<br>al 2017	East<br>Asia<br>China	58yr<br>M	Unknown	S-LAM	Cough<br>Pneumonia	CT: high-density<br>nodules (in the lungs)	Lung tissue<br>pathology	Pneumonia	Pneumonectomy	Alive and well<br>38 mo later
	East<br>Asia<br>China	64yr<br>M	Unknown	S-LAM	Asymptomatic	CT: high-density<br>nodules (in the lungs)	Lung tissue<br>pathology	Angiomatosis	Pneumonectomy	Alive and well<br>37 mo later
	East<br>Asia<br>China	41yr<br>M	Unknown	S-LAM	Cough<br>Chronic bronchitis	CT: high-density<br>nodules (in the lungs)	Lung tissue<br>pathology	NA	Pneumonectomy	Alive and well<br>30 mo later
	East<br>Asia<br>China	79yr<br>M	Unknown	S-LAM	Cough<br>Chronic bronchitis	CT: high-density<br>nodules (both lungs)	Lung tissue<br>pathology	Angiomatosis	Pneumonectomy	Alive and well<br>23 mo later

Table S1 (Continued)

Author(reference)<br>Year	Country	Age<br>Sex	Smoking	Form	Presentation	Imaging presentation	Diagnostic technology	Misdiagnosed disease	Treatment	Course
Wang et al<br>2017	East<br>Asia<br>China	50yr<br>M	Unknown	S-LAM	Asymptomatic	CT: high-density<br>nodules (both lungs)	Lung<br>tissue<br>pathology	Lung cancer	Pneumonectomy	Alive and well<br>21 mo later
Chen et al<br>2017	East<br>Asia<br>China	55yr<br>M	Unknown	S-LAM	Dyspnoea<br>Cough<br>Pneumothorax(n>3)	CT: diffuse cystic<br>lesions (both lungs)<br>2-20mm in the diameter	Symptom<br>CT imaging	Chronic bronchitis	Sirolimus	Alive and well<br>in the short term
Yamanaka et al<br>2017	East<br>Asia<br>Japan	34yr<br>M	Current	TSC-LAM	Pneumothorax(n>3)<br>Dyspnoea	CT: diffuse cystic<br>lesions (both lungs)<br>over 0.5 in the diameter	Lung biopsy<br>pathology	NA	NA	Alive 10 mo later
Meilan et al<br>2017	North<br>Americal<br>USA	48yr<br>M	Never	S-LAM	Pneumothorax(n=2)<br>Renal AMLs	Diffuse cystic<br>lesions (both ungs)	Lung tissue<br>pathology	NA	NA	Lost to follow-up
Wang et al<br>2018	East<br>Asia<br>China	22yr<br>M	Unknown	S-LAM	Abdominal pain	CT: thin-walled cysts<br>(rightlung)diffuse<br>abdominal cysts	Mass biopsy<br>pathology	Acute appendicitis	Abdominal<br>lumpectomy	Alive in the short<br>term
Liu et al<br>2018	East<br>Asia<br>China	66yr<br>M	Current	TSC-LAM	Cough and<br>Hemoptysis	CT: high-density nodules<br>(in the lungs)	Thoracoscopy<br>pathology	Lung cancer	Pneumonectomy	Alive 6 mo later
Yalikun et al<br>2019	East<br>Asia China	28yr<br>M	Unknown	TSC-LAM	Asymptomatic	MRI: recurrent mass<br>(right temporal lobe)	Thoracoscopy<br>pathology	Angiomatosis	Resection of a<br>brain mass	Lost to follow-up

Table S1 (Continued)

Author(reference) Year	Country	Age Sex	Smoking	Form	Presentation	Imaging presentation	Diagnostic technology	Misdiagnosed disease	Treatment	Course
Kabi et al 2020	South Asia India	18yr M	Unknown	TSC-LAM	Pneumothorax(n=2)	CT: diffuse cystic lesions (both lungs)	Thoracotomy pathology	NA	Sirolimus	Alive in the short term

Abbreviations: Yr, year; M, male; LAM, lymphangioleiomyomatosis; TSC, tuberous sclerosis complex; S-LAM, Sporadic; AMLs, angiomyolipomas; Mo, Month. CT, computed tomography;
